# Supplementary material for: Clinical and Microbiological Impact of Implementing a Decision Support Algorithm through Microbiologic Rapid Diagnosis in Critically Ill Patients: An Epidemiological Retrospective Pre-/Post-Intervention Study
Source: Biomedicines. 2023 Dec 16;11(12):3330. doi: 10.3390/biomedicines11123330 (PMC10741655; doi:10.3390/biomedicines11123330)
Supplement: Supplementary file 1 [file biomedicines-11-03330-s001.zip › biomedicines-2739353-supplementary.pdf]

Clinical and Microbiological Impact of Implementing a Decision Support Algorithm through Microbiologic Rapid Diagnosis in Critically Ill Patients: An Epidemiological Retrospective Pre-/Post-Intervention Study

Supplementary material

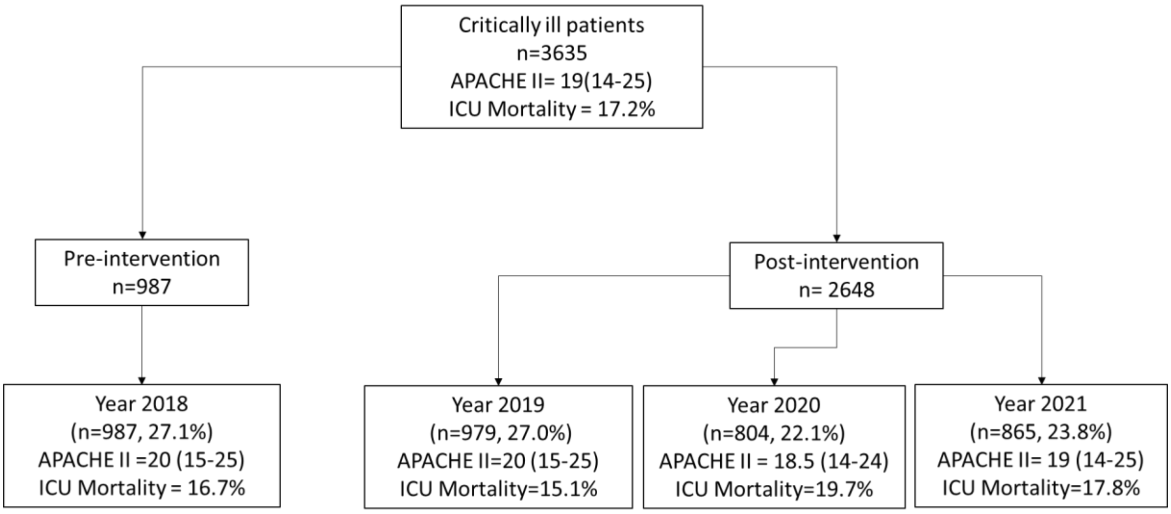

Figure S1. Flowchart of patients included in the study.

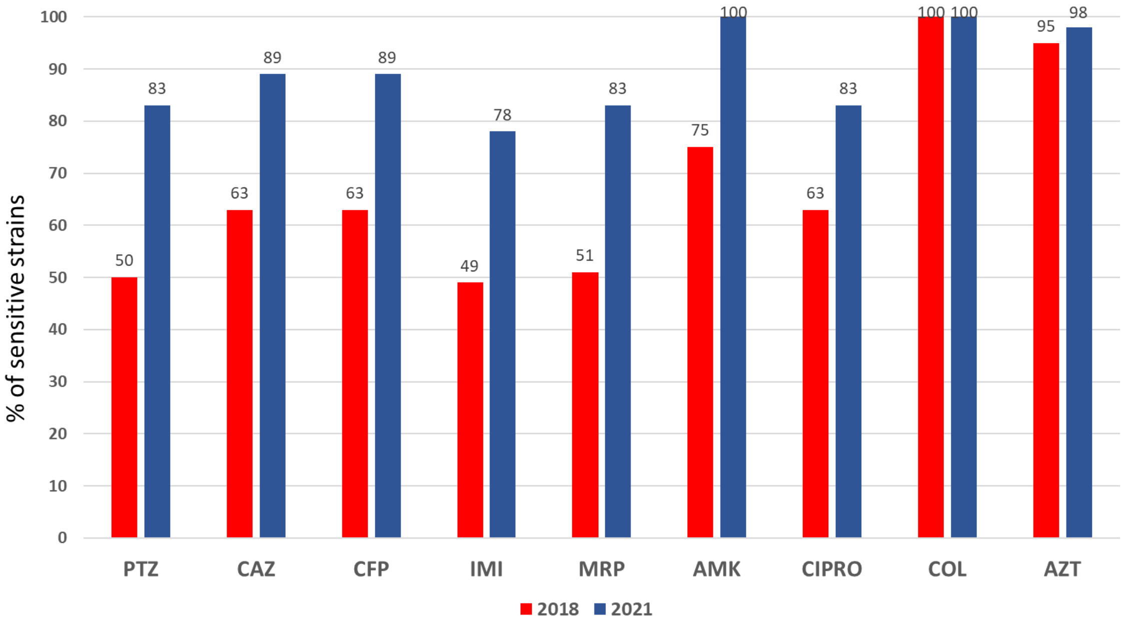

Figure S2. Resistance pattern of Pseudomonas aeruginosa strains in isolates from respiratory samples for the main antimicrobials comparing pre (2018) and post-intervention periods (2021). PTZ= Piperacillin/Tazobactam ; CAZ= Ceftazidime; CFP= Cefepime ; IMI= Imipenem; MRP= Meropenem; AMK= Amikacine; CIPRO= Ciprofloxacin ; COL= Colistin; AZT=Aztreonam.

Table S1. Risk Factors for Multidrug-Resistant Pathogens according to IDSA guidelines (1)

- Prior intravenous antibiotic use within 90 days
- Septic shock at time of VAP
- ARDS preceding VAP
- Five or more days of hospitalization prior to the occurrence of VAP
- Acute renal replacement therapy prior to VAP onset

(1)Andre C. Kalil, Mark L. Metersky, Michael Klompas, John Muscedere, Daniel A. Sweeney, Lucy B. Palmer et al. Management of Adults With Hospital-acquired and Ventilator-associated Pneumonia: 2016 Clinical Practice Guidelines by the Infectious Diseases Society of America and the American Thoracic Society. Clinical Infectious Diseases 2016;63(5):e61–111

Definitions

Frequency measures

The definitions and calculation formula are those of the National Epidemiological Registry of Intra-ICU Acquired Infection (ENVIN-HELICS). https://hws.vhebron.net/envin-helics/

Incidence rates (IR) were calculated for each of the controlled infections expressed in relation to the number of patients at risk or the number of days at risk.

In all cases, the numerator includes the absolute number of cases of the infection analysed and the denominator uses 1000 days of exposure to risk or ICU stay.

- Incidence density(id) of invasive mechanical ventilation-associated pneumonia:

VAP id= Number of cases of pneumonia in MV X 1,000 / total days of mechanical ventilation.

- Incidence density(id) of Catheter-related bacteraemia (CRB) and bacteraemia of unknown origin (BUNK).

CRB/BUNK id= Number of CRB and BUNK episodes x 1,000 / total catheter days

- Incidence density (id) of Secondary bacteraemia (SB)

SB id= Number of SB episodes x 1,000/ total ICU days

- Incidence density Catheter Associated Urinary Tract Infection (CAUTI)

CAUTI id= Number of CAUTI episodes x 1,000 / total urinary catheter days

Table S2. Characteristic of 3635 critically ill patients included according to the study period.

Study Period	Pre-intervention	Intervention	p-value
Variable	2018 (n=987)	2019-21(n=2648)	
Demographics and severity			
Age, mean (Q1-Q3)	64 (52-73)	63 (50-73)	**0.02**
Male, n (%)	634 (64.2)	1716 (64.8)	0.78
APACHE, mean (Q1-Q3)	20 (15-25)	19 (14-25)	**0.04**
SOFA, mean (Q1-Q3)	3.0 (2.0-5.1)	2.9 (2.0-5.1)	**0.008**
Patients type			
Surgical, n (%)	275 (27.9)	680 (25.7)	0.19
Medical, n (%)	712 (72.1)	1968 (74.3)	0.18

COVID-19 within medical patients, n (%)	0 (0.0)	398 (20.2)	**NA**
Comorbidities			
Obesity, n (%)	123 (12.4)	446 (26.8)	**<0.001**
Diabetes, n (%)	239 (24.2)	586 (22.1)	0.19
Chronic heart disease, n (%)	61 (6.2)	100 (3.8)	**0.002**
COPD, n (%)	126 (12.7)	274 (10.3)	**0.04**
Chronic Rennal failure, n (%)	93 (9.4)	246 (9.3)	0.95
Immunosupression, n (%)	43 (4.3)	116 (4.4)	1.0
Laboratory			
Hemoglobin g/dL, median (Q1-Q3)	10.3 (8.6-12.2)	10.0 (8.5-11.9)	**0.003**
WBC count 10^3/uL, median (Q1-Q3)	10.8 (8.1-13.9)	10.4 (8.1-13.9)	0.18
Serum creatinine mg/dL, median (Q1-Q3)	0.7 (0.6-1.1)	0.7 (0.6-1.1)	0.05
RCP mg/dL, median (Q1-Q3)	9.9 (5.3-18)	9.1 (4.7-16.6)	**0.006**
Microbiologically confirmed infections during ICU stay			
Total number of infections, n (%)	83 (8.4)	380 (14.3)	**<0.001**
Ventilator-associated pneumonia (VAP)*, n(%)	21 (25.3)	131 (34.5)	0.06
Bacteraemia secondary to other septic foci (BS), n(%)	17 (20.0)	40 (10.5)	**0.01**
Bacteraemia of unknown origin (BUNK), n (%)	9 (10.8)	60 (15.8)	0.25
Catheter-associated urinary tract infection (CAUTI), n(%)	8 (9.6)	42 (11.0)	0.70
Ventilator-associated tracheobronchitis (VAT), n (%)	5 (6.0)	32 (8.4)	0.46
Catheter-related bacteraemia (CRB), n (%)	4 (4.8)	47 (12.3)	**0.04**
Intra-abdominal infections (IAI), n (%)	4 (4.8)	6 (1.6)	0.08
Skin and soft tissue infection (SSTI), n (%)	4 (4.8)	5 (1.3)	0.09
Others, n (%)	11(13.2)	6 (1.6)	**<0.001**
Main micro-organisms isolated during ICU stay			
Total number of microorganisms isolated, n (%)	102 (10.3)	500 (18.9)	**<0.001**
Staphylococcus aureus	16 (15.7)	70 (14.0)	0.7
Escherichia coli	13 (12.7)	49 (9.8)	0.5
Klebsiella pneumoniae	10 (9.8)	58 (11.6)	0.7
Pseudomonas aeruginosa	9 (8.8)	78 (15.6)	**0.04**
Enterobacter aerogenes	7 (6.8)	12 (2.4)	**0.02**
Serratia marcescens	6 (5.8)	20 (4.0)	0.55
Haemophilus influenzae	5 (4.9)	23 (4.6)	1.0
Enterococcus faecium	4 (3.9)	9 (1.8)	0.33
Klebsiella oxytoca	4 (3.9)	11 (2.2)	0.50
Proteus mirabilis	3 (2.9)	8 (1.6)	0.60
Citrobacter spp.	3 (2.9)	13 (2.6)	1.0
Enterobacter cloacae	3 (2.9)	29 (5.8)	0.35
Enterococcus faecalis	3 (2.9)	30 (6.0)	0.31
Others	16 (15.6)	90 (18.0)	0.67
Incidence density of reported ICU-associated infections			
VAP episodes/1000 mechanical ventilation days (95% CI)	5.5 (3.5-8.3)	7.3 (6.1-8.7)	0.66
CAUTI episodes /1000 urinary catheter days (95% CI)	1.3 (0.6-2.6)	1.6 (1.1-2.1)	0.62
BRC and BUNK episodes / 1000 catheter days (95% CI)	1.7 (0.3-6.6)	2.8 (2.2-3.7)	0.89
BS episodes / 1000 ICU days (95% CI)	2.3 (1.4-3.7)	1.3 (1.0-1.8)	0.81
Complications and Outcome			
Invasive Mechanical ventilation , n(%)	425 (43.1)	1377 (52.0)	**<0.001**
LOS ICU, mean (Q1-Q3)	4.0 (2.0-8.0)	3.9 (2.1-7.9)	**0.009**
Crude ICU Mortality, n (%)	165 (16.7)	460 (17.4)	0.67

Table S3. Microorganisms isolated in patients with ventilator-associated pneumonia according to the study period.

	**Pre-intervention**	**Intervention**		
**Year**	**2018**	**2019**	**2020**	**2021**
**N° Patients/ N° Microorganisms**	21 /27	12/20	38/56	92/132
Staphylococcus aureus n (%)	9 (33.3)	4 (20.0)	15 (26.8)	28 (21.0)
Klebsiella spp n (%)	4 (14.8)	----	7 (12.5)	16 (12.1)
Escherichia coli n (%)	3 (11.1)	3 (15.0)	3 (5.3)	12 (9.0)
Pseudomonas aeruginosa n (%)	3 (11.1)	4 (20.0)	9 (16.0)	24 (18.2)
Haemophilus influenzae n (%)	2 (7.4)	3 (15.0)	3 (5.3)	6 (4.5)
Proteus mirabilis n (%)	1 (3.7)	----	2 (3.5)	2 (1.5)
Stenotrophomona maltophilia n (%)	1 (3.7)	----	1 (1.8)	2 (1.5)
Streptococcus pneumoniae n (%)	1 (3.7)	2 (10.0)	1 (1.8)	4 (3.0)
Enterobacter spp. n (%)	1 (3.7)	----	2 (3.6)	12 (9.0)
Acinetobacter spp. n (%)	----	1 (5.0)	1 (1.8)	3 (2.3)
Staphylococcus aureus methicillin resistant n (%)	----	1 (5.0)	----	2 (1.5)
Serratia marcescens	----	----	3 (5.3)	6 (4.5)
Aspergillus spp	----	----	----	7 (5.3)
Others	2 (7.4)	2 (10.0)	9 (16.0)	8 (6.0)

Table S4. ATB consumption expressed in defined daily doses (DDD) according to the pre- and post-intervention period. Rate Ratio (RR) shows the variation of DDD between the periods compared.

	**2018(1)**	**2019(2)**	**2020(3)**	**2021(4)**	**RR 2 vs 1 (95%CI)**	**RR 3 vs 1 (95%CI)**	**RR 4 vs 1 (95%CI)**
**Overall**	128.7	124.2	113.8	66.0	0.96 (0.93-0.99)*	0.88 (0.80-0.91)*	0.51 (0.49-0.53)*
**MRP**	21.8	25.9	19.6	16.0	1.18 (1.11-1.27)*	0.89 (0.84-0.96)*	0.73 (0.68-0.78)*
**PTZ**	7.42	6.19	6.89	2.96	0.83 (0.73-0.94)*	0.92 (0.82-1.03)	0.39 (0.34-0.45)*
**CAZ**	2.37	0.99	1.63	0.65	0.41 (0.31-0.54)*	0.68 (0.55-0.84)*	0.27 (0.20-0.35)*
**CFP**	1.06	0.60	0.31	0.09	0.56 (0.39-0.75)*	0.29 (0.22-0.49)*	0.08 (0.03-0.2)*
**AZT**	0.02	0.72	1.25	1.33	36.0 (7.1-180.0)*	62.5 (12.0-309.0)*	66.5 (13.2-329.3)*

MRP= Meropenem; PTZ= Piperacillin/Tazobactam ; CAZ= Ceftazidime CFP= Cefepime; AZT= Aztreonam. * p-value <0.05
